# Effectiveness of Text Message Reminders on Adherence to Inhaled Therapy in Patients With Asthma: Prospective Multicenter Randomized Clinical Trial

**DOI:** 10.2196/12218

**Published:** 2021-02-09

**Authors:** Carlos Almonacid, Carlos Melero, Antolín López Viña, Carolina Cisneros, Luis Pérez de Llano, Vicente Plaza, Juan Luis García-Rivero, Auxiliadora Romero Falcón, Jacinto Ramos, Teresa Bazús González, María Andrés Prado, Alfonso Muriel

**Affiliations:** 1 Department of Respiratory Medicine Instituto Ramón y Cajal de Investigación Sanitaria University of Alcala de Henares Madrid Spain; 2 Department of Respiratory Medicine Hospital Universitario 12 de Octubre, Institute for Health Research (i+12) Complutense University of Madrid Madrid Spain; 3 Department of Respiratory Medicine Hospital Universitario Puerta de Hierro Autonoma University of Madrid Majadahonda Spain; 4 Department of Respiratory Medicine Hospital Universitario La Princesa Autónoma University of Madrid Madrid Spain; 5 Department of Respiratory Medicine Hospital Universitario Lucus Augusti University of Lugo Lugo Spain; 6 Department of Respiratory Medicine Hospital de la Santa Creu i Sant Pau, Institut d’Investigació Biomédica Sant Pau Autònoma University of Barcelona Barcelona Spain; 7 Department of Respiratory Medicine Laredo Hospital Laredo Spain; 8 Department of Respiratory Medicine Hospital Universitario Reina Sofia University of Cordoba Córdoba Spain; 9 Department of Respiratory Medicine Hospital Universitario de Salamanca University of Salamanca Salamanca Spain; 10 Department of Respiratory Medicine Hospital Universitario Central de Asturias University of Oviedo Oviedo Spain; 11 Department of Health Information Management Fundación Jimenez Diaz Madrid Spain; 12 Unit of Clinical Biostatistics Instituto Ramón y Cajal de Investigación Sanitaria, Consorcio Centro de Investigación Biomédica en Red de Epidemiología y Salud Pública University of Alcala de Henares Madrid Spain

**Keywords:** asthma, adherence, SMS, control, cell phone, inhaler, Smartinhaler

## Abstract

**Background:**

Poor adherence to inhaled medication in asthma patients is of great concern. It is one of the main reasons for inadequate asthma control.

**Objective:**

The goal of the research was to determine if motivational messages using short message service (SMS, or text) improved adherence to inhaled medication in patients with asthma.

**Methods:**

A prospective multicenter randomized parallel-group clinical trial was conducted in 10 asthma clinics in Spain. Adherence was assessed with electronic monitors (Smartinhaler, Adherium Ltd) connected to inhalers. Patients in the SMS group received psychologist-developed motivational messages every 3 days for 6 months.

**Results:**

There were 53 patients in the SMS group and 88 patients in the control group. After 6 months, mean electronic adherence was 70% (SD 17%) in the intervention group and 69% (SD 17%) in the control group (*P*=.82). Significant differences between the study groups in morning and evening adherence to inhaled therapy, asthma control, exhaled nitric oxide levels, or improvement of lung functions were not observed.

**Conclusions:**

Motivational messages were not useful to improve adherence to inhaled asthma medication compared with usual care.

## Introduction

Epidemiological studies show a high prevalence of poor asthma control [1-3]. Poor adherence is one of the most frequent causes of this problem [2-18]. The measurement of adherence to inhalers is a complicated task. Different methods have been used in daily practice, including clinical judgment, response to treatment, generally validated self-report questionnaires [19], or specifically designed instruments such as the Test of Adherence to Inhalers [20,21]. All these methods tend to overestimate treatment adherence [22,23]. Exhaled nitric oxide (FeNO) measurement has been proposed as an objective technique to measure treatment compliance [24]. In the last 30 years, electronic monitoring devices have been developed with this proposal [25-28]. These devices can record the time and number of doses taken and remind the patient to take medication.

Adherence to inhaled therapy depends on multiple factors. Asthma education programs plus information and communication technologies could be more useful to improve adherence than using them separately. All cell phones can send or receive short message service (SMS, or text) messages, which are a convenient, widely used mode of communication [29-32]. It has been shown that text messages improve appointment adherence [10], but very little research has focused on the effect of messages to reinforce adherence using SMS.

Several studies reviewed the effect of reminders on asthma control and adherence to treatment [33-40]. Only a few studies have used electronic devices to assess adherence to inhalers. In these studies, reminders were also sent through the same electronic device (SmartTrack or Smartinhaler [both Adherium Ltd]) that measured adherence to treatment, not through SMS [39,40]. Other studies used self-report questionnaires to measure inhaler adherence, but this leads to decreases the quality of the results. To date, a benefit in adherence to treatment from motivational text messages sent to a cell phone has not been reported.

The objective of this study is to measure the effect of motivational messages designed by a psychologist on treatment adherence compared with usual care in patients with moderate to severe asthma.

## Methods

### Trial Design

We conducted a parallel-group randomized controlled trial in patients with moderate or severe asthma at 10 university hospitals in Spain. Patients were recruited from 2013 to 2014. Patients were randomized to an SMS group or a control group; follow-up was for 6 months.

### Participants

The eligibility criteria for participants were (1) aged 18 to 85 years, (2) asthma diagnosed according to the Global Initiative for Asthma (GINA) criteria [41], (3) all patients treated with maintenance therapy according GINA guidelines, (4) currently own cell phone, (5) not currently being treated with systemic corticosteroids or biologic drugs, (6) not currently participating in another research study.

Exclusion criteria were (1) inability to use inhaler devices [30]; (2) other associated chronic respiratory disease (eg, chronic obstructive pulmonary disease); (3) previous participation in a study using SMS-related asthma reminders (4) asthma exacerbation within 3 months of inclusion in the study (defined by oral corticosteroid use, emergency department visit, or hospitalization); (5) previous treatment with budesonide/formoterol as maintenance and reliever therapy; (6) other uncontrolled severe medical conditions.

### Interventions

The SMS group received psychologist-developed motivational messages every 3 days for 6 months in addition to usual care recommendations according to GINA guidelines [41]. SMS messages were not reminders to take a dose of medication. The control group was treated with general care recommendations alone according to GINA guidelines [41]. Four study visits were required (1 for enrollment and 3 for follow-up) in both groups. Outcome data were collected at the clinic by study staff in V0, V1 (1 month later), V3 (3 months later), and V6 (6 months later—end of study).

#### Medications and Inhaler Monitoring

**Figure 1:**
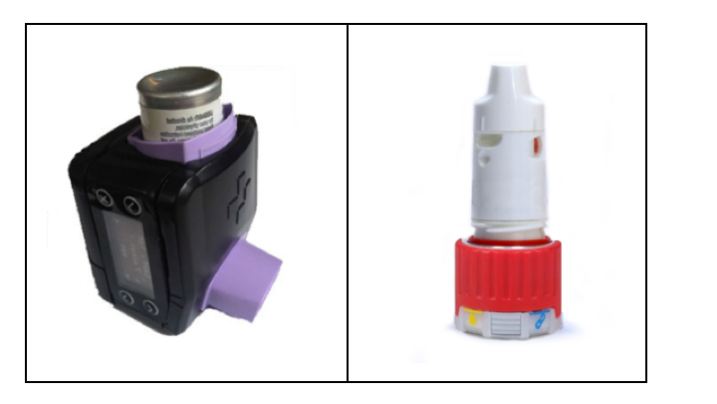
SmartTrack devices connected to pressurized metered-dose inhaler (left) and Turbuhaler inhalation device (right).

All patients received a SmartTrack device (Smartinhaler [Adherium Ltd]; Figure 1) that clipped on their ICS/LABA inhaler. SmartTrack is an electronic device that allows measuring adherence to the inhalers. Each puff is recorded in the device memory. SmartTrack records the date and time the inhaler was used and the number of puffs taken. SmartTrack features include reminders, onscreen questions about asthma control, and medication feedback viewing online, but these have been deactivated. After the device was activated during the enrollment (visit 0), it recorded the date and time of all maneuvers to measure adherence to treatment. The data were uploaded to a secure cloud server with local backup to the investigator’s computer.

Patients were treated with an ICS/LABA inhaler for maintenance therapy (fluticasone propionate/salmeterol Accuhaler/Diskus inhaler, budesonide/formoterol Turbuhaler inhaler, or beclomethasone/formoterol metered-dose inhaler). They also used albuterol pressurized metered-dose inhaler as rescue medication. Device reliability and precision have been reported according to other publications [38].

#### SmartTrack Training and Monitoring

Electronic monitoring via the Smartinhaler was used to assess adherence to inhaled asthma therapy. All patients received brief instructions on the use of the SmartTrack. Patients with adherence higher than 80% were considered regular adherents. Electronic monitors were attached to participant controller medications in the SMS and control groups.

#### Text Messages

Patients assigned to the intervention group only received SMS communications about the importance of asthma medication every 3 days for 6 months; messages were not reminders to take a dose of medication. The messages, written by a psychologist in collaboration with pulmonologists, were randomly switched every 3 days and include the following:

- Remember that performing the treatment of inhaled medication keeps your asthma controlled.
- Inhaled medication helps to maintain asthma control.
- Remember to take inhaled medication; it keeps you well.
- Have you taken your inhaled medication?
- Do you take your inhaled medication in the morning and the evening?
- Maybe now is the time to take your inhaled medication.
- Remember to take inhaled medication as prescribed by your doctor.

#### Asthma Education

All patients were provided an asthma action plan on the first day written in accordance with the GINA guidelines. Inhaler technique was reviewed, and any problems were corrected. At each visit, inhaler technique and the asthma action plan were reviewed.

#### Asthma Control

Asthma control was measured using the Asthma Control Test (ACT) [42], and a score ≥20 identified well-controlled asthma patients.

#### Pulmonary Function

Spirometry with bronchodilator test was performed according to the European Respiratory Society/American Thoracic Society (ERS/ATS) guidelines [43]. Values for the Mediterranean population were used [44]. Forced expiratory volume in 1 second (FEV1) pre and postbronchodilator were measured.

#### Fractional Exhaled Nitric Oxide

An FeNO level was measured before spirometry using the equipment available in each center. The test was performed following the ERS/ATS recommendations [45].

### Outcomes

Demographic data, ACT, lung function (spirometry), FeNO levels, and exacerbation history in the previous year were collected at the enrollment visit (V0). ACT, lung function, FeNO levels, and exacerbation history were also collected at V1, V3, and V6.

The primary outcome was adherence to inhaled medication. SmartTrack devices were used to quantify adherence to control medication. The secondary outcomes were asthma control measured by the ACT, spirometry parameters (FEV1, forced vital capacity, and FEV1/forced vital capacity ratio), FeNO levels, number of asthma exacerbations, visits to the emergency department, and hospital admissions due to asthma.

### Sample Size

The sample size was calculated according to an adherence rate to inhaled asthma therapy between 30% and 70% [9]. On the assumption of the maximal uncertainty of 50% and considering a clinically relevant difference of 25%, with a type I error of .05, a power of 80%, and a 20% loss, 73 patients per arm were needed (total 146 patients).

### Randomization, Blinding, and Allocation Concealment

We generated the 2 comparison groups using simple randomization with an equal allocation ratio by referring to a table of random numbers. A mathematician, also in charge of randomization of the SMS sending, generated the sequences of random allocation, who enrolled participants, and who assigned participants to interventions and SMS sending.

Blinding of patients was not possible. To avoid bias and with ethics approval, physicians were not notified about the SmartTrack recording function or SMS intervention until the study ended.

### Statistical Methods

Categorical variables were expressed as frequencies and percentages and continuous variables as means and standard deviations. The chi-square or Fisher exact test were used for the analysis of categorical variables, and the Student *t* test or Mann-Whitney *U* test were used for the comparison of quantitative variables according to the standard or nonnormal distribution of variables. Tests were 2-tailed. Statistical significance was set at *P*<.05. Statistical analyses were performed using the SPSS Statistics version 20.0 (IBM Corp).

### Ethics Approval and Trial Registration

The clinical research ethics committees of the participating centers approved the study. All participants provided written informed consent. The study was classified by the Spanish Agency of Medicines and Medical Devices as a noninterventional imposed postauthorization safety study, and for this reason we were not required to register the clinical trial.

## Results

### Patient Participants

Of patients recruited, 63.8% (97/152) were women. At the first data collection time point, the mean age was 48.9 (SD 16.7) years, and the mean duration of asthma was 16.5 (SD 13) years. A total of 6.6% (10/152) of patients were current smokers. Most patients were well controlled at enrollment, with a mean ACT score of 20.2 (SD 4.5) points. The mean FEV1 before the bronchodilation test was 79% (SD 17.9%) predicted, the mean FEV1 after bronchodilation test was 82.5% (SD 16.2%) predicted, and the mean FeNO level was 37.9 (SD 35.9) ppb. A total of 13.2% (20/152) of patients reported one or more exacerbations during the last 12 months, 15.8% (24/152) reported an episode of asthma-related urgent health care utilization and prednisone use in the previous year, and 5.9% (9/152) required admission to hospital.

**Figure 2:**
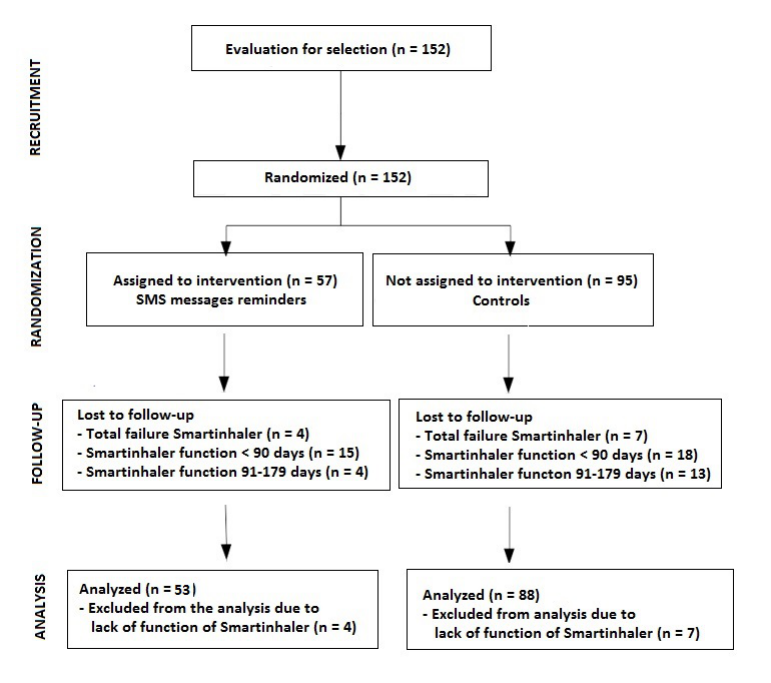
Flow diagram of the progress through the phases of a parallel randomized trial of two groups.

A failure with the randomization program unbalanced the number of patients in both arms. During the study period, 57 patients were assigned to the intervention group, and 95 to the control group. At follow-up, 11 patients were excluded because of the total failure of the electronic monitoring device. The final analysis included 53 patients in the intervention group and 88 in the control group. The flowchart of the study population is shown in Figure 2. Demographic and baseline characteristics were similar between groups, apart from a lower proportion of men and university education in the SMS group (Table 1).

**Table 1:** Clinical characteristics of patients at baseline.

Variable			SMS^a^ group (n=57)		Control group (n=95)		*P* value
Gender, male, n (%)			15 (26)		40 (42)		.05
Age in years, mean (SD)			50.4 (16.4)		48 (16.9)		.40
**Education level, n (%)**							
	Primary/secondary studies	47 (82)		61 (64)		.40	
	University degree	7 (12)		20 (21)		.40	
**Working status, n (%)**							
	Unemployed	6 (11)		10 (11)		.79	
	Employed	34 (60)		54 (57)		.79	
	Housewife	4 (7)		11 (12)		.79	
	Retired	13 (23)		18 (19)		.79	
**Smoking history, n (%)**							
	Current smoker	4 (7)		6 (6)		.75	
	Ex-smoker	17 (30)		34 (36)		.75	
	Never smoker	36 (63)		55 (58)		.75	
Self-management education program, n (%)			10 (18)		16 (17)		.91
**Asthma history, mean (SD)**							
	Years from diagnosis	16.6 (11.6)		16.5 (14.3)		.98	
	Years from starting treatment	13.3 (10.3)		13.2 (12.1)		.10	
	Years from follow-up in outpatient clinics	7.2 (7)		6.8 (7.8)		.78	
**Inhaler type, n (%)**							
	Accuhaler/Diskus	18 (32)		38 (40)		.57	
	Turbuhaler	26 (46)		37 (39)		.57	
	pMDI^b^	13 (23)		20 (21)		.57	
**Inhalation technique, correct steps, n (%)**							
	Acceptable	40 (70)		75 (79)		.22	
	Unacceptable	17 (30)		20 (21)		.22	
**Asthma-related events in the previous 12 months, mean (SD)**							
	Exacerbations	0.6 (1.2)		0.4 (0.8)		.21	
	Emergency department visits	0.2 (0.6)		0.2 (0.7)		.79	
	Hospital admission	0.05 (0.2)		0.04 (0.2)		.76	
Asthma Control Test, mean (SD)			20.3 (4.2)		20.0 (4.8)		.69
FeNO^c^ level, ppb, mean (SD)			42.2 (37.5)		33.7 (34.3)		.18
FEV1^d^ before bronchodilation test, % predicted, mean (SD)			79.0 (17.5)		79.5 (18.4)		.92
FEV1 after bronchodilation test, % predicted, mean (SD)			79.6 (16.7)		85.3 (15.7)		.16

^a^SMS: short message service.

^b^pMDI: pressurized metered-dose inhaler.

^c^FeNO: exhaled nitric oxide.

^d^FEV1: forced expiratory volume in 1 second.

### Adherence to Treatment

#### Primary Outcome

Adherence data were recorded throughout the study starting from enrollment, and 92.8% (141/152) of patients had analyzable SmartTrack data. No adherence data were available for 7.2% (11/152) of patients due to device failure; 21.7% (33/152) of SmartTracks monitored adherence for at least 90 days, 11.2% (17/152) operated fine for between 90 and 179 days, and 59.9% (91/152) worked precisely until the study ended.

The data transmission software that communicated with the Smartinhaler platform failed throughout the study in most of the participating centers since it did not work with the hospital proxy servers. Due to this failure, the backup files generated during each visit were used for the analysis. Differences between the study groups were not found in SmartTrack failures (*P*=.51).

After 6 months, the mean electronic adherence was 70% (SD 17%) in the intervention group and 69% (SD 17%) in the control group (*P*=.82). Adherence to inhalers was higher in both groups in the morning (control group 79% [SD 19%] vs 60% [SD 24%] in the afternoon and SMS group 80% [SD 23%] vs 59% [SD 29%] in the afternoon; Table 2).

**Table 2:** Results at the end of the study.

Variables			SMS^a^ group (n=53)		Control group (n=88)		*P* value	
Adherence %, mean (SD)			70 (17)		69 (17)		.82	
Morning adherence %, mean (SD)			80 (23)		78 (19)		.44	
Evening adherence %, mean (SD)			59 (29)		60 (24)		.75	
Asthma Control Test score, mean (SD)			21.6 (3.7)		22.1 (3.5)		.46	
FeNO^b^ level, ppb^c^, mean (SD)			36.5 (35.5)		28.3 (22.0)		.14	
FEV1^d^ before bronchodilation test, % predicted, mean (SD)			77.3 (16.2)		80.9 (18.0)		.42	
FEV1 after bronchodilation test, % predicted, mean (SD)			85.9 (12.4)		84.5 (16.2)		.73	
**Exacerbation episodes** **, n (%)**								.22
	0	37 (84)		68 (92)		—		
	1	7 (16)		5 (7)		—		
	≥2	0		1 (1)		—		
**Visits to emergency department, n (%)**								.43
	0	40 (91)		71 (95)		—		
	1	4 (10)		4 (5)		—		
	≥2	0		0		—		
Hospital admission for asthma, n (%)			0		0		—	

^a^SMS: short message service.

^b^FeNO: exhaled nitric oxide.

^c^ppb: parts per billion.

^d^FEV1: forced expiratory volume in 1 second.

#### Secondary Outcomes

At the end of the study (V6), no differences were found between the SMS and control group in ACT, FEV1, FeNO levels, exacerbations, emergency department visits, or hospital admissions (Table 2).

## Discussion

### Principal Findings

In this study, motivational SMS was not associated with an improvement in adherence to inhaled medication. We also found no significant differences in secondary variables, asthma control, lung function, exhaled nitric oxide levels, or exacerbations due to asthma.

Education strategies are useful to improve adherence to asthma therapy [10]. Few studies have examined the effect of SMS reminders on adherence to asthma treatment. Strandbygaard et al [46] conducted a 12-week study with 26 asthma patients randomized to the SMS group (n=12) or control group (n=14). The SMS group received this message daily: “Remember to take your asthma medication morning and evening. From the Respiratory Unit.” A medicine dose-count for Diskus inhaler was used to assess adherence. The mean adherence rate increased in the SMS group and decreased in controls, with a difference in mean adherence rate between the 2 groups after 12 weeks of 17.8% (95% CI 3.2%-32.3%; *P*=.02). Changes in adherence in the SMS group, however, were not associated with clinical or functional improvement. Few patients were included in this study, and adherence may have been overestimated due to the possibility to take actions before the medical visit.

In another study, Charles et al [47] used the audiovisual reminder function on the Smartinhaler metered-dose inhaler device. After 12 weeks, the absolute difference in the median percentage of medication taken between the intervention (n=55) and control (n=55) groups was 18% (95% CI 10%-26%; *P*<.001). But, again, no improvements in clinical or lung functions were observed.

In a 6-month cluster-randomized study, Foster et al [35] assessed the effect of inhaler reminders plus adherence feedback (n=21) versus usual care or personalized discussion with the general practitioners (n=22) in the primary care setting. Reminders were associated with improvements in adherence (73% [SD 26%] vs 46% [SD 28%] of prescribed daily doses, *P*<.001) as well as with an improvement in the ACT but without differences between the study groups.

In a recent systematic review of interventions aimed at improving adherence to inhaled corticosteroids for asthma, 11 studies assessing electronic trackers or reminders versus control were analyzed [10]. Electronic trackers or reminders led to better adherence of 19 percentage points (95% CI 14.47-25.26; 6 studies; moderate-quality evidence), but a clear benefit on clinical outcomes was not found.

### Limitations

In our study, a simple intervention based on motivational SMS sent every 3 days for 6 months was not adequate to improve adherence. Different reasons may account for this adverse finding. First, the messages sent were motivational; they were not reminders for taking medication. Second, the pattern of nonadherence behavior (eg, erratic, deliberate, unwitting) [21] was not evaluated nor were patients stratified according to a nonadherent model. Third, asthma was well controlled at baseline as shown by high ACT scores and lung function parameters; the magnitude of improvement depends to a large extent on the degree of control of the disease before the intervention.

An interesting finding of this study, in which all patients had twice-daily medication schedules, is that adherence rates were higher in the morning than in the evening. It is possible that in patients on single-dose inhaled medication regimens, SMS could have had a more significant impact on adherence.

Although the Smartinhaler is accurate in recording and retaining actuation data [48,49], potential malfunction during real-life use by patients is possible. In our study, failure of communication technology occurred in all centers without differences between the study groups; in these cases, however, analyses were performed using backup files. Only 91% (SD 59.9%) of the Smartinhalers worked fine and collected information until the study ended.

In our study, patients were recruited in specialized asthma units established in different hospitals throughout Spain for the management of patients with severe and difficult-to-control asthma [50]. Therefore, it is possible that this particular circumstance may account for the adequate adherence at baseline and proper control of asthma before enrollment and at the end of the study.

### Conclusions

In summary, in this prospective randomized clinical study, reinforcement of adherence to inhaled asthma medication using motivational SMS via cell phones was not capable of improving adherence. Mobile health interventions are increasingly popular for implementing on a large scale in different chronic health conditions [51-53], but further research into these issues is needed. Despite the negative results that may be justified because of the study’s limitations as described above, the study’s results are not conclusive. However, an interesting fact that can be seen in both groups is that morning adherence to the inhaler is much higher than afternoon adherence. These data should be verified in a new well-designed clinical trial that could reinforce the use of ultralong action treatments, which only need to be taken once a day, in order to improve the adherence and control of asthma.

## Abbreviations

ACT: Asthma Control Test
ERS/ATS: European Respiratory Society/American Thoracic Society
FeNO: exhaled nitric oxide
FEV1: forced expiratory volume in 1 second
GINA: Global Initiative for Asthma
ICS/LABA: inhaled combination of corticosteroids and a long-acting beta 2 agonist
SMS: short message service
